# Galium

**Published:** 1868-01

**Authors:** D. L. Phares

**Affiliations:** Newtonia, Miss.


### ARTICLE IV.

*Galium.* By D. L. Phares, M. D., Newtonia, Miss.

This genus probably derives its name from the Greek *gala*, (milk,) one or more species, the G. verum especially, having been much used in ancient as well as modern times for flavoring, coloring and curdling milk intended for cheese, and hence called also cheese-rennet. Or the generic name may have come in another way from *gala*, the G. Aperine being used, as Dioscorides tells us, by the shepherds of his time as a filter to strain milk through, and in Sweden for the same purpose, as stated by Linnæus. The specific naeae of this last is from *apairo*, (to lay hold of,) the scrbo us stems, hirsute leaves and hooked, bristly fruit well adapting it to seizing and taking hairs out of the milk, and at the same time giving cause for the common names, cleavers, catch-weed, scratch-weed, &c., the fruit adhering to whatever it comes in contact with. This property of adhering to the clothes and person led the Greeks to call it *philanthropon*, man-lover. Being much relished by geese, it has received the name goose-grass also. The name bed-straw is doubtless from the old form of the verb to strew, strow, straw, one of the species being much used in former times by thrifty English dames to strew their beds with, thus imparting a pleasant fragrance.

This genus belongs to the order Rubiaceæ, and has many properties, both medicinal and economical, in common with the madder. The flowering stems, with alum, dye yellow, and the roots red. These plants are used by the Indians for dying feathers, porcupine quills, &c. At the instance of the British Council of Trade, they were once cultivated like madder, and yielded *fourteen hundred pounds of roots to the acre.* In passing, I cannot permit the loss of this opportunity to say, that as they grow luxuriantly in the Southern States, they might be cultivated with much profit by some of our enterprising farmers, thus varying our crops and increasing our prosperity.

Like madder, they color the bones of birds and other animals feeding on them. The G. tuberosum, we are told, is cultivated in China for the roots, which are cooked whole, or after being reduced to meal; and they are esteemed salubrious. The G. Aperine and some of the other species have been for ages regarded as "purifiers of the blood," and used in the spring with that view in broths. The juice also has been long in use, both in Europe and America, for scrofula, scurvy, freckles, lepra, and cutaneous eruptions generally. In these affections the infusion or expressed juice is taken internally and applied locally. Scrofulous and other tumors are said to be promptly discussed by taking the juice internally and applying the bruised plant locally. Several species have considerable reputation in the treatment of hysteria and epilepsy, as the G. verum formerly, the G. palustre in the latter affection specially and recently in France, and in America the G. trifidum, (G. tinctorium, L.) The galiums are said to be very valuable also in erysipelas, scarlatina, congestion of the spleen, spitting of blood, dysentery, and for dissolving or crumbling down urinary calculi. I have no experience with them in any of the above affections.

My use of them has been in another line of practice, in which I have employed them, in many cases, with very decided benefit—especially the G. Alperine, which, however, does not grow in this latitude. It is found in the more northern of the Southern States. There are seven other species growing in the Southern States, most of them widely diffused. Nearly all possess some medicinal properties in common, in greater or less degree; but as they differ in other respects, they should be carefully studied. As they are all rare in my vicinity, I have not had opportunities of observing and determining the special properties of each.

As strong heat dissipates the medicinal properties of the galiums, we may thus account for the fact that some practitioners claim good results, while others pronounce them inert, having used inadvertently a worthless decoction. Cold or warm water extracts the active principle. It may be given in the form of expressed juice, if fresh f℥ss, if inspissated, ʒi., or infusion, f℥ij.—iv., daily, in chronic or subacute cases; every hour or two in those that are acute, grave and attended with much febrile excitement. The infusion is made of the bruised, dry plant, ℥ iv., or of the green, *ad libitum*, and water Oij. The water is better warm if the vessel is closely covered till cold.

I have used the galiums in suppression of the urinary secretion, in many cases of inflammation of the kidneys, bladder and urethra, and in several cases of dropsy, with very satisfactory results. The preparations I have used proved powerfully diuretic and refrigerant. In cases of great debility, or a feeble circulation, it might even prove dangerous to exhibit the medicine in large doses, unless cautiously guarded to prevent too much refrigerating effect. A year or two ago I had occasion to prescribe it for a lady suffering very severely with acute nephritis. She complained so much of chilliness being induced by it, that I had difficulty in prevailing on her to continue its use in diminished doses.

It also possesses sedative properties, but how great I cannot now positively state. It is a very good remedy in acute gonorrhœa and prostatitis; and by analogy I would infer for acute inflammations generally of the mucous surfaces.

Although I know nothing of its alleged property of dissolving urinary calculi, I can very well understand how a careless observer might be deceived into the belief. Its soothing, relaxing influence, together with a copious secretion of a bland urine, is capable of removing small concretions from the kidneys and bladder, causing them to pass through the ureters and urethra without resistance of the muscles being excited. I can understand also why and how it might be beneficial in some hemorrhages, dysentery, scarlatina and other fevers, some cases of erysipelas, and other affections. But of these I cannot now affirm anything. Speaking of calculi, in some cases of hæmaturia, especially in connection with pertussis, I have, under the use of soothing diuretics, seen the blood soon disappear and, then in twenty-four hours, two or three hundred calculi discharged with the urine; the calculi varying in size from that of a large grain of sand to that of a small pea. But in these I did not suspect the breaking down of larger calculi, nor do I yet believe there was anything of the kind.

